# Heterotopic Pancreas in the Gallbladder: Case Report and Literature Review

**DOI:** 10.1155/2021/6611291

**Published:** 2021-01-30

**Authors:** Emad Aborajooh, Ibrahim Khalil Khairi Ghayada, Yasser Mustafa Issa Lafi

**Affiliations:** ^1^Department of Surgery and Anaesthesia, Faculty of Medicine, Mutah University, Kerak, Jordan; ^2^Department of Medicine, Al-Basheer Hospital, Amman, Jordan

## Abstract

**Introduction:**

Heterotopic pancreas (HP) is the congenital presence of pancreatic tissue outside its normal location in the absence of vascular and anatomical connection with the main pancreas. HP can affect any part of the gastrointestinal tract, and it is mostly encountered in the stomach. The gallbladder is a rare site of HP, and our literature review revealed that only 38 cases were reported. We present a case of HP in the gallbladder that was presented with a picture of acute cholecystitis. After the case presentation, we will discuss HP in the gallbladder by reviewing the literature. *Case Presentation*. A 49-year-old male presented to the emergency department complaining of progressively worsening right upper abdominal pain for the last 24 hours. After thorough history and physical examination, a provisional diagnosis of acute cholecystitis was made. Abdominal ultrasonography revealed a rim of edema surrounding the gallbladder wall with two stones impacted at the gallbladder neck. Laparoscopic cholecystectomy was performed with an uneventful postoperative course, and the patient was discharged the next day. Microscopic examination of the gallbladder showed that a heterotopic pancreatic tissue, composed of a large number of pancreatic acini and few ducts with the absence of islets of Langerhans, was found around the cystic duct. The patient was asymptomatic at the regular follow-up six months postoperatively.

**Conclusion:**

HP in the gallbladder is an extremely rare finding. Its clinical presentation is not different from other cholecystopathic diseases. Most cases were accompanied by cholelithiasis. Preoperative laboratory and imaging modalities are usually not helpful in the diagnosis of HP in the gallbladder. The definitive diagnosis is made by histological examination of the gallbladder specimen. Laparoscopic cholecystectomy is sufficing treatment.

## 1. Introduction

Heterotopic pancreas (HP) is the congenital presence of pancreatic tissue outside its normal location in the absence of vascular and anatomical connection with the main pancreas [1]. There is no consensus about the exact origin of HP until the present. Three theories have been proposed to explain it. One adopted that HP is formed as a result of pancreatic tissue separation during the embryonic rotation [2]. Another theory suggests that the longitudinal growth of the intestines is responsible for the migration of some cells from pancreatic buds which causes HP in different regions [2]. Irregularity in the notch signaling system is a recent accepted theory, which believes that irregularity in the signaling system that decides the destiny of pancreatic cells, specifically abnormalities in Hes-1 (Hairy/enhancer of split), might be a contributor in the formation of HP in the gallbladder [3]. HP can be seen anywhere along the gastrointestinal tract, and it is mostly encountered in the stomach, duodenum, and colon with percentages of 27.5%, 25.5%, and 15.9%, respectively [4]. Also, it can be seen in the esophagus, Meckel`s diverticulum, gallbladder, biliary tract, spleen, liver, omentum, and lung. Usually, heterotopic pancreatic tissue is asymptomatic, but it can present with nonspecific gastrointestinal symptoms. In autopsy studies, HP was found in about 0.5%–13.7% patients [4]. The estimated incidence of HP in abdominal surgeries is 0.2% [5]. HP in the gallbladder is an extremely rare finding. To the best of our knowledge, there are only 38 cases in the literature. In this case report, we present a case of HP in the gallbladder and the current literature reviewed.

## 2. Methodology and Data Collection

A thorough search among the published literature using the PubMed search engine was done. Specific search terms (keywords) were heterotopic pancreas or ectopic pancreas and gallbladder. The following filters were used: English language and human and case reports. A bibliography of relevant articles was reviewed in an attempt to find other relevant articles. The following data were collected: demographic information including age and gender, clinical presentation, sonographic findings including presence of gallstones, surgical intervention, site and size of the heterotopic pancreas (HP), and histological type of HP.

### 2.1. Case Report

A 49-year-old male presented to the emergency department complaining of progressively worsening right upper abdominal pain, which was continuous, dull aching in nature, and associated with vomiting for the last 24 hours. The patient had a previous history of mild abdominal pain, especially with fatty meals, for the past six months. On examination, he had a heart rate of 85 beats/minute, a blood pressure of 140/85 mmHg, a temperature of 37.4°C, and a respiratory rate of 16 breaths/minute. The abdominal examination revealed localized right upper quadrant tenderness with a positive Murphy's sign. Laboratory investigations showed an elevated WBC count of 16.7 ∗ 103/*μ*L (reference range: 4.5–10 ∗ 10^3^/*μ*L) with 77% neutrophils, hemoglobin was 14.6 g/dL (reference range: 14–18 g/dL), and platelet count was 193 ∗ 10^3^/*μ*L (reference range: 150–450 ∗ 10^3^/*μ*L). Other laboratory results include aspartate aminotransferase (AST) was 35 U/L (reference range: up to 37 U/L), alanine aminotransferase (ALT) was 33 U/L (reference range: up to 42 U/L), alkaline phosphatase was 103 U/L (reference range: 98–279 U/L), total bilirubin was 0.82 mg/dL (reference range: up to 1 mg/dL), direct bilirubin was 0.29 mg/dL (reference range: up to 0.20 mg/dL), and serum amylase was 97 U/L (reference range: 40–140 U/L). Urea, creatinine, and serum electrolytes (sodium and potassium) were within normal limits. Sonographic examination showed a thin gallbladder wall with two stones (12 and 4 mm) impacted in the neck, a rim of edema surrounding the gallbladder wall, and a normal common bile duct.

Laparoscopic cholecystectomy was performed. The operative findings were an edematous gallbladder wall with an adherent omentum to it, difficult dissection around the cystic duct, and only one single (15 mm) stone identified in the gallbladder specimen. The postoperative course went uneventfully, and the patient was discharged home the next day.

**Figure 1:**
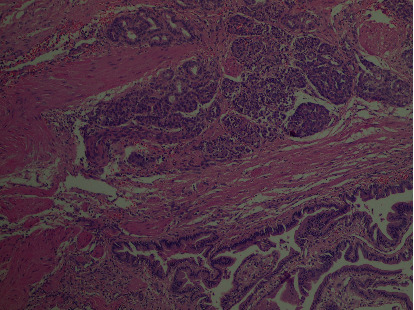
H & E stain 40x: presence of pancreatic tissue (few ducts and large number of acini with absence of islets), i.e., type II, around the cystic duct.

Microscopic examination of the gallbladder demonstrated an acute on the top of chronic cholecystitis with cholesterolosis. Moreover, a heterotopic pancreatic tissue, composed of a large number of pancreatic acini and few ducts with the absence of islets of Langerhans, was found around the cystic duct (Figure 1). Follow-up investigations after two weeks, including complete blood count, AST, ALT, alkaline phosphatase, total bilirubin, direct bilirubin, serum amylase, and lipase, were all normal. The patient was asymptomatic at regular follow-up six months postoperatively.

### 2.2. Literature Review

A thorough search in the literature shows only 38 cases that have been reported with HP in the gallbladder. We made a summary table of reported cases found in the literature (Table 1). Female predominance was noted, with an M : F ratio of about 1 : 2. However, our patient was male. The ages of reported cases ranged from eight to eighty with a mean of 43.6 and a median of 46, and most cases were above the age of 40. In most cases, biliary cholecystopathic symptoms were the primary indication for surgical interventions. Cholelithiasis was present in about 50% of cases. Moreover, HP in the gallbladder may resemble gallbladder polyps or malignancy by imaging studies, and this was the indication for surgery in about 10% of reported cases.

Out of the 38 cases, 29 were classified using Heinrich's classification, which revealed that the most common type is type I. The cases were distributed as follows: 69% were type I, 24% were type II, while type III was present in 7% of cases. Our case was type II. This is the 39^th^ case in the literature.

## 3. Discussion

In the eighteenth century, Jean Schultz et al.'s study was the first one which described heterotopic pancreas. Classification of the ectopic pancreas was made by Heinrich et al. into three types [4]:

- Type I: mimicking the normal pancreatic tissues with the presence of ducts, acini, and endocrine islets
- Type II: the presence of a few ducts, a large number of acini, and the absence of endocrine islets
- Type III: the presence of a large number of ducts, a few numbers of acini, and the absence of endocrine islets

This classification was modified later by Fuentes in 1973 to include four types of HP [35]:

- Type I: Resembles the normal pancreatic tissue with the presence of ducts, acini, and endocrine islets
- Type II: Canalicular variant with pancreatic ducts
- Type III or exocrine pancreas only
- Type IV or endocrine pancreas only

Macroscopically, HP tissue could appear as an exophytic polypoid growth or as yellow-coloured nodules, and it could measure from few millimetres to four centimetres [20]. HP in the gallbladder is extremely uncommon. A case series of 212 cases of HP performed by Mayo Clinic revealed only one case of HP in the gallbladder [20]. While the male to female ratio in HP in the gastrointestinal system is 3 : 1 [36], our literature review showed that HP in the gallbladder has a female predominance properly due to the higher number of cholecystectomies among female patients.

Although HP in the gallbladder is mostly asymptomatic and found incidentally postoperatively [5], the literature review showed that HP is associated with cholecystopathetic symptoms in most of the reported cases. The presentations range from biliary colic to hydrops gallbladder and even perforation of the gallbladder with peritonitis [5]. The ectopic tissue is prone to the same pathological conditions as the normal pancreatic tissue such as abscess, pseudocyst formation, or chronic pancreatitis [37]. Despite that it was not reported in the literature about HP in the gallbladder, the malignant changes of the gastric heterotopic pancreas have been described [37]. Asahi Sato et al. found a direct association between the developments of cholecystopathetic symptoms and the level of pancreatic enzymes in the bile [30].

In most cases, the preoperative imaging modalities such as ultrasound or computed tomography were not helpful in the diagnosis of HP in the gallbladder as HP can resemble cholesterol polyps, adenomyoma, carcinoma, cholecystitis, cysts, abscess formation, and other lesions [20].

The value of considering HP in the differential diagnosis of the gallbladder disease is arguable since cholecystectomy is usually performed as a treatment of cholecystopathetic symptoms or to rule out malignancy.

## 4. Conclusion

HP in the gallbladder is an extremely rare finding. Its clinical presentation is not different from other cholecystopathic diseases. Most cases are accompanied by cholelithiasis. Preoperative laboratory and imaging modalities are usually not helpful in the diagnosis of HP in the gallbladder. The definitive diagnosis is made by histological examination of the gallbladder specimen. Laparoscopic cholecystectomy is sufficing treatment.

## Figures and Tables

**Table 1:** Demographic data, clinical presentation, and histopathology of 38 cases of heterotopic pancreas in the gallbladder.

Author	Age	Sex	Preoperative presentation	GBS	Procedure	Site	Type
Al-Shraim [6]	39	M	Chronic cholecystitis	No	L.C.	GB wall	NS
Basrur [7]	40	F	Biliary colic	Yes	L.C.	GB wall	NS
Beltrán [8]	22	F	Biliary colic	Yes	L.C.	Cystic duct	Type 1
Beltrán [8]	8	M	Biliary colic	Yes	L.C.	Infundibulum	Type 1
Bhana [9]	47	F	Acute cholecystitis	No	L.C.	Cystic duct	Type 1
Cerullo [10]	51	F	Acute cholecystitis	Yes	L.C.	NS	Type 2
Cerullo [10]	53	F	Chronic cholecystitis	No	L.C.	Fundus	Type 3
Ben-Baruch [11]	45	M	Inflamed gallbladder leads to perforation and peritonitis	Yes	NS	Neck	Type 1
Hadzi-Nikolov [12]	48	F	NS	NS	NS	GB wall	Type 1
Elfiving [13]	59	F	NS	Yes	NS	NS	Type 1
Elhence, P [14]	18	F	Chronic cholecystitis	Yes	L.C.	Neck	Type 2
Elpek [5]	40	M	Acute cholecystitis	No	NS	Neck	Type 1
Ferhatoglu [15]	39	M	Chronic cholecystitis with polyp	No	L.C.	Body	NS
Ferhatoglu [15]	48	F	Chronic cholecystitis with polyp	No	L.C.	Fundus	NS
Foucault [16]	72	F	Cancer	Yes	O.C.	Posterior wall	NS
Gucer [17]	80	M	Acute cholecystitis	No	L.C.	Body	Type 1
Inceoglu [18]	55	F	Hydrops GB biliary colic	Yes	NS	Cystic duct	Type 2
Kantor [19]	18	F	Acute cholecystitis	NS	NS	NS	NS
Klimis [20]	35	M	Acute cholecystitis	No	L.C.	Body	Type 2
Kondi-Paphiti [21]	58	F	Chronic cholecystitis	No	L.C.	Neck	NS
Kondi-Paphiti [21]	48	F	Chronic cholecystitis	Yes	L.C.	NS	NS
Kondi-Paphiti [21]	53	F	Cancer	Yes	O.C.	NS	Type 1
Koukourakis [22]	31	F	Chronic cholecystitis	NS	L.C.	GB wall	Type 1
Koukourakis [22]	36	F	Chronic cholecystitis	NS	L.C.	GB wall	Type 1
Limaiem [23]	55	M	Chronic cholecystitis	Yes	L.C.	Neck	Type 3
Mboti [24]	23	F	Chronic cholecystitis	No	L.C.	Fundus	Type 2
Meshikhes [25]	23	F	Biliary colic	Yes	L.C.	Fundus	Type 1
Mrak [26]	75	F	NS	Yes	L.C.	Cystic duct	Type 1
Murakami [27]	49	F	Gallbladder polyp	No	L.C.	NS	Gastric and type 2 HP
Qizilbash [28]	54	M	Pancreatitis	No	NS	GB wall	Type 1
Sanchiz Cárdenas [29]	43	M	Acute cholecystitis	Yes	L.C.	GB wall	NS
Sato [30]	60	F	Asymptomatic	No	L.C.	Neck	Type 2
Sharma SP [31]	43	M	Chronic cholecystitis	Yes	L.C.	Neck	Type 1
Shiwani [1]	20	F	Biliary colic and jaundice	Yes	L.C.	Outside GB	Type 1
Sroczynski [32]	55	M	Pancreatitis	Yes	L.C. converted to O.C.	Fundus	Type 1
Vidgoff [33]	52	M	NS	No	NS	NS	Type 1
Weppner [3]	26	F	Acute cholecystitis	Yes	L.C.	Neck	Type 1
Won lee [34]	36	F	Segmental adenomyomatosis by CT	No	L.C.	Cystic duct	Type 1
Our case	49	M	Acute cholecystitis	Yes	L.C.	Cystic duct	Type 2

M: male, F: female, GBS: gallbladder stone, L.C.: laparoscopic cholecystectomy, NS: not specified, O.C.: open cholecystectomy, and GB: gallbladder.

## Data Availability

All data supporting the findings of this study are available within the article.
